# Benchmarking Relatedness Inference Methods with Genome-Wide Data from Thousands of Relatives

**DOI:** 10.1534/genetics.117.1122

**Published:** 2017-07-26

**Authors:** Monica D. Ramstetter, Thomas D. Dyer, Donna M. Lehman, Joanne E. Curran, Ravindranath Duggirala, John Blangero, Jason G. Mezey, Amy L. Williams

**Affiliations:** * Department of Biological Statistics and Computational Biology, Cornell University, Ithaca, New York 14853; † South Texas Diabetes and Obesity Institute, University of Texas Rio Grande Valley, Brownsville, Texas 78520; ‡ Department of Medicine, University of Texas Health San Antonio, San Antonio, Texas 78229; § Department of Genetic Medicine, Weill Cornell Medicine, New York, New York 10065

**Keywords:** relatedness estimation, identical by descent, admixture

## Abstract

Inferring relatedness from genomic data is an essential component of genetic association studies, population genetics, forensics, and genealogy. While numerous methods exist for inferring relatedness, thorough evaluation of these approaches in real data has been lacking. Here, we report an assessment of 12 state-of-the-art pairwise relatedness inference methods using a data set with 2485 individuals contained in several large pedigrees that span up to six generations. We find that all methods have high accuracy (92–99%) when detecting first- and second-degree relationships, but their accuracy dwindles to <43% for seventh-degree relationships. However, most identical by descent (IBD) segment-based methods inferred seventh-degree relatives correct to within one relatedness degree for >76% of relative pairs. Overall, the most accurate methods are Estimation of Recent Shared Ancestry (ERSA) and approaches that compute total IBD sharing using the output from GERMLINE and Refined IBD to infer relatedness. Combining information from the most accurate methods provides little accuracy improvement, indicating that novel approaches, such as new methods that leverage relatedness signals from multiple samples, are needed to achieve a sizeable jump in performance.

## Abstract (precis)

Relatedness inference is an essential component of many genetic analyses and popular in consumer genetic testing. Ramstetter *et al.* evaluate twelve.....

THE recent explosive growth in sample sizes of genetic studies has led to an increasing proportion of individuals with at least one close relative in a data set, necessitating relatedness detection. As the number of pairs in a sample grows quadratically in its size, for a constant rate of relatedness among pairs, proportionately more individuals will have close relatives in large data sets. This pervasiveness has relevance to nearly every genetic analysis performed in moderate to large-scale data, including trait mapping and population genetics. In particular, inferring relatedness between samples (Weir *et al.* 2006; Thompson 2013; Speed and Balding 2015) is essential to avoid spurious signals in genetic association studies (Marchini *et al.* 2004; Hirschhorn and Daly 2005; Voight and Pritchard 2005); empowers linkage analysis by enabling the correct specification of pedigree structures (O’Connell and Weeks 1998; Ott 1999; Epstein *et al.* 2000); facilitates identification of relatives in the context of forensic genetics (Jobling and Gill 2004; Weir *et al.* 2006; Kayser and de Knijff 2011); and is needed to account for or remove relatives in population genetic analyses (Queller and Goodnight 1989; Hurst 2009; Schraiber and Akey 2015). Relatedness estimation has also drawn the interest of the general public via companies that offer genetic testing services and advertise their ability to find customers’ relatives, thus allowing individuals to explore their ancestry and genealogy. The broad utility of relatedness detection has motivated the development of numerous methods for such inference. These methods work by estimating the proportion of the genome shared identical by descent (IBD) between individuals (Weir *et al.* 2006; Speed and Balding 2015) or a closely related quantity, where an allele in two or more individuals’ genomes is said to be IBD if those individuals inherit it from a recent common ancestor (Thompson 2013). Characterizing the true relatedness of two or more samples is challenging for several reasons, including chance sharing of alleles between individuals who are only distantly related, and the fact that the distributions of IBD proportions for different relatedness classes overlap (Hill and Weir 2011; Thompson 2013) (*e.g.*, first cousins and half-first cousins).

Motivated by the substantial need to identify relatives in modern samples, we present an evaluation of 12 state-of-the-art pairwise relatedness methods, each capable of scaling to analyze thousands of individuals, including seven that directly infer genome-wide relatedness measures (Manichaikul *et al.* 2010; Thornton *et al.* 2012; Li *et al.* 2014; Moltke and Albrechtsen 2014; Sun and Dimitromanolakis 2014; Chang *et al.* 2015; Conomos *et al.* 2016) and five IBD segment detection methods (Gusev *et al.* 2009; Browning and Browning 2011a, 2013a,b; Durand *et al.* 2014) that we used to infer these quantities. To assess these methods, we used SNP array genotypes from Mexican American individuals contained in large pedigrees from the San Antonio Mexican American Family Studies (SAMAFS) (Mitchell *et al.* 1996; Duggirala *et al.* 1999; Hunt *et al.* 2005). Our analysis sample included 2485 individuals genotyped at 521,184 SNPs (Supplemental Note in File S1) within pedigrees that span up to six generations, and with genotype data from as many as five generations of individuals. Given this large sample, including 13 pedigrees with >50 individuals (Supplemental Material, Figure S1 in File S1), numerous relatives exist, and we used these to evaluate the inference methods. Specifically, we analyzed >3700 pairs of individuals within each of the first- through fifth-degree relatedness classes, 816 and 73 sixth- and seventh-degree relatives, respectively, and >3 million pairs of individuals that are reported as unrelated (Table 1). Prior evaluations of relatedness inference methods included only a subset of the methods we evaluate, and either considered simulated data (Manichaikul *et al.* 2010; Thornton *et al.* 2012; Moltke and Albrechtsen 2014; Sun and Dimitromanolakis 2014; Conomos *et al.* 2016) (which may not fully capture the complexities of real data), used small sample sizes (Manichaikul *et al.* 2010; Huff *et al.* 2011; Thornton *et al.* 2012; Conomos *et al.* 2016), or did not consider sixth- and seventh-degree relatives (Manichaikul *et al.* 2010; Thornton *et al.* 2012; Moltke and Albrechtsen 2014; Conomos *et al.* 2016). This analysis of real data from large numbers of up to sixth-degree relatives, as well as dozens of seventh-degree relative pairs, provides a comprehensive evaluation of existing pairwise relatedness inference methods.

**Table 1:** Numbers of pairs of individuals in the SAMAFS data set that passed sample filters*^a^* and are reported to have relatedness between first- and seventh-degree or as unrelated

Degree	Number of pairs
1	4969
2	6625
3	8241
4	7636
5	3794
6	816
7	73
Unrelated	3,051,598
Total	3,083,752

We combined reported monozygotic (MZ) twins with the set of first-degree relatives.

a Supplemental Note in File S1.

The performance metric for this study is the rate at which each method infers the pairs of samples to have the same degree of relatedness as that reported in the SAMAFS pedigrees. These reported relationships are generally reliable, and we filtered out relative pairs whose degree of relatedness is potentially inflated due to cryptic relatedness between their ancestors (Supplemental Note in File S1). Some programs infer the degree of relatedness (Li *et al.* 2014), while others infer a kinship coefficient (Manichaikul *et al.* 2010; Thornton *et al.* 2012; Moltke and Albrechtsen 2014) or a coefficient of relatedness (Chang *et al.* 2015; Conomos *et al.* 2016) [which is two times the kinship coefficient (Wright 1922)], and the remainder instead detect IBD segments (Gusev *et al.* 2009; Browning and Browning 2011a, 2013a,b; Durand *et al.* 2014) (Table 2). To infer the degree of relatedness from an estimated kinship coefficient, we used the mapping recommended in the KING paper (Table S1 in File S1), which is generally consistent with simulations (Manichaikul *et al.* 2010).

**Table 2:** Properties of the 12 relationship inference methods we analyzed

Method	Version	Citation	Type	Output	Parallelized?	Runtime (× cores if > 1) [× number of runs]	Requires independent markers	Input required from outside program	Accounts for population structure
ERSA	2.0	Li *et al.* (2014)	IBD segment-based	Degree of relatedness	N	14.3 + 96.3 hr (×16)*^a^*	N	IBD segments	NA
fastIBD	Beagle 3.3.2	Browning and Browning (2011a)	IBD segment-finding	IBD segments	N	55.2 hr [× 10]	N	NA	NA
GERMLINE (-haploid)	1.5.1	Gusev *et al.* (2009)	IBD segment-finding (distinguishes IBD1 and IBD2)	IBD segments	N	19.2 min + 96.0 hr (×16)*^b^*	N	Phased genotypes	NA
HaploScore	NA	Durand *et al.* (2014)	IBD segment-based	IBD segments	N	2.4 + 96.3 hr (×16)*^a^*	N	IBD segments; phased genotypes	NA
IBDseq	r1206	Browning and Browning (2013a)	IBD segment-finding	IBD segments	Y	33.1 hr (×16)	N	NA	NA
KING (KING-robust)	1.4	Manichaikul *et al.* (2010)	Allele frequency-based IBD estimate	IBD 0,1,2 proportions	N	4.6 min	Y	NA	Y
PC-Relate	2.0.1	Conomos *et al.* (2016)	Allele frequency-based IBD estimate	IBD 0,1,2 proportions	N	8.9 hr + 4.6 min*^c^*	Y	Pairwise kinship coefficients	Y
PLINK 1.9	1.90b2k	Chang *et al.* (2015)	Allele frequency-based IBD estimate	IBD 0,1,2 proportions	N	18.1 sec	Y	NA	N
PREST-plus	4.1	Sun (2012)	Allele frequency-based; uses linkage model	IBD 0,1,2 proportions	N	178.9 hr	N	NA	N
REAP	1.2	Thornton *et al.* (2012)	Allele frequency-based IBD estimate	IBD 0,1,2 proportions	N	3.8 + 2.8 hr*^d^*	Y	Ancestral population allele frequencies; sample ancestry proportions	Y
Refined IBD	Beagle 4.1	Browning and Browning (2013b)	IBD segment-finding (distinguishes IBD1 and IBD2)	IBD segments	Y	96.0hr (× 16) [× 3]	N	NA	NA
RelateAdmix	0.1	Moltke and Albrechtsen (2014)	Allele frequency-based IBD estimate	IBD 0,1,2 proportions	Y	15.8 hr (×16) + 2.8 hr*^d^*	Y	Ancestral population allele frequencies; sample ancestry proportions	Y

Type indicates the inference methodology the program uses. Runtime is wall clock time to run the program with any additional time to run programs needed for input as indicated. We ran parallelized programs using the numbers of cores indicated in parentheses, and ran fastIBD and Refined IBD multiple times as recommended by the authors, with counts indicated in square brackets. Input required from outside program indicates extraneous information needed to run the program. Programs that use either principal components, sample ancestral population proportions, or that use a model designed for multiple populations are indicated as accounting for population structure. “Y” indicates yes, “N” indicates no, and “NA” indicates not applicable. Runtimes are from a machine with four AMD Opteron 6176 2.30 GHz processors (64 cores total) and 256 GB memory.

a Additional time to phase the data using Beagle 4.1 and run GERMLINE.

b Additional time to phase the data using Beagle 4.1.

c Additional time to obtain KING relatedness estimates; base PC-Relate time is the sum of time to run this method and PC-AiR (Conomos *et al.* 2015).

d Additional time to obtain ancestral population proportions using ADMIXTURE (Alexander *et al.* 2009).

For IBD detection methods that report the number of IBD segments shared at a locus (Gusev *et al.* 2009; Browning and Browning 2013b), denoted IBD0, IBD1, and IBD2 for the corresponding number of copies that are IBD, it is straightforward to calculate a kinship coefficient (Thompson 2013). This coefficient, $\varphi_{ij},$ between a pair of samples i,j denotes the probability that a randomly selected allele in individual *i* is IBD with a randomly selected allele from the same genomic position in individual j. Let $k_{ij}^{\left(0\right)},$ $k_{ij}^{\left(1\right)},$ and $k_{ij}^{\left(2\right)}$ denote the proportion of their genomes that individuals i,j share IBD0, IBD1, and IBD2, respectively; then the kinship coefficient is $\varphi_{ij}=\frac{k_{ij}^{\left(1\right)}}{4}+\frac{k_{ij}^{\left(2\right)}}{2}.$ The proportions $k_{ij}^{\left(1\right)}$ and $k_{ij}^{\left(2\right)}$ are simply the sum of the genetic lengths of the IBD1 and IBD2 segments, respectively, between samples i,j divided by the total genetic length of the genome analyzed. For the IBD detection methods (Browning and Browning 2011a, 2013a; Durand *et al.* 2014) that do not distinguish between regions that are IBD1 from IBD2, the proportion of the genome that is inferred to be IBD0 provides an alternate means of estimating the degree of relatedness (Table S1 in File S1), with the ranges of values here again from the KING paper (Manichaikul *et al.* 2010). We classified pairs of individuals with lower kinship coefficients or higher IBD0 rates than indicated for the eighth-degree range as unrelated.

The results from the analysis are shown in Figure 1, which depicts the proportion of sample pairs inferred to be within each of the degree classes that we considered (first- through eight-degree and unrelated), separated according to their reported relatedness degree. All methods perform well when inferring first- and second-degree relatives, with accuracies ranging from 98.8 to 99.5% for first-degree relatives, and from 92.8 to 98.6% for second-degree relatives. However, the methods’ accuracies diverge for more distant relatedness, with the IBD segment-based methods generally having higher accuracy than those that rely on allele frequencies of independent markers. For example, for sixth- and seventh-degree relatives, the top-performing IBD segment-based method has 58.1 and 42.5% accuracy, respectively, while the highest performing allele frequency-based method has an accuracy of only 44.6 and 27.4%, respectively. This general pattern applies to fourth- and fifth-degree relatives as well, although with less discrepancy between these two inference approaches for these closer relatives. The decreased inference accuracy of all methods for higher relatedness degrees is likely due to the exponential drop in mean pairwise IBD shared and an increased coefficient of variation for more distant relationships (Hill 1993; Visscher 2009; Hill and Weir 2011).

**Figure 1:**
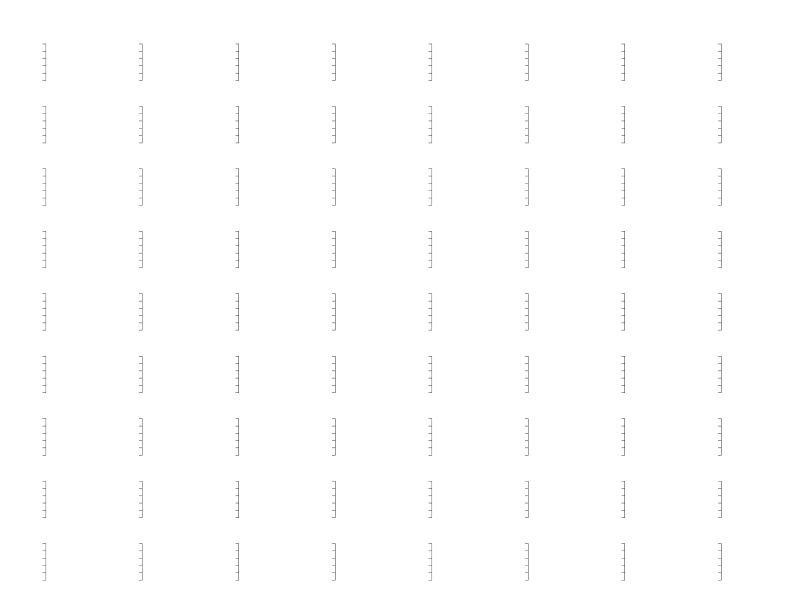
Performance comparison of the evaluated methods using the SAMAFS data set. Bar plots denote the percentage of sample pairs that are reported to have a given degree of relatedness and that are inferred to be related as the indicated degree. The bar plots are separated on the horizontal axis by the reported relatedness degree and on the vertical axis by inferred relatedness degree. For clarity, the plots list above each bar the inferred percentage that the corresponding bar depicts. Program names listed in red are IBD segment-based methods while those in black use allele frequencies for inference. Red horizontal bars under a bar plot indicate that the corresponding inferences agree with the reported relationships.

While the accuracies for exact inference of distant relatives are fairly low among all methods, the IBD segment-based methods (excluding fastIBD) are correct to within one degree of the reported relationship at a rate of ≥95.3% for sixth-degree relatives and ≥76.7% for seventh-degree relatives. At the same time, ERSA, GERMLINE, and Refined IBD classify ≥80.4% pairs of unrelated individuals correctly, and several other methods also correctly infer ∼80% pairs of unrelated individuals, although many of these methods perform poorly when classifying reported relatives. The inference of ∼20% of the >3 million unrelated samples as eighth-degree or closer relatives suggests the presence of a nontrivial fraction of unreported relationships in these data. Alternatively, and perhaps more likely, many of these may be false positive relationships, as distinguishing pairs of unrelated individuals from fairly distant relatives is difficult. With the lower bound for eighth-degree relatives being a total of 19.5 cM of IBD segments shared between individuals, spurious inferences at this level are possible, with IBD segments detected in regions subject to historical selection (Albrechtsen *et al.* 2010) or with low SNP density potentially leading to inflated IBD proportions. In that regard, we note that some analyses of IBD reweight segments that overlap regions with excess IBD sharing to improve the reliability of overall sharing rates (Browning and Browning 2013c; Ball *et al.* 2016). Additionally, analyses that consider relatedness among the parents and/or children of inferred distant relatives have the potential to avoid some of these issues, and indeed, the recently developed relatedness classification method PADRE does analyze familial relatedness signals and shows improved accuracy (Staples *et al.* 2016).

Overall, the most accurate programs for first- through seventh-degree and unrelated classification are ERSA, GERMLINE, and Refined IBD—all IBD segment-based methods. The improved accuracy of these methods may be due to their focus on identifying long stretches of identical haplotype segments that more readily discriminate recent shared relatedness from chance sharing of alleles. The IBDseq method, while performing well for inferring first- through seventh-degree relatives, infers a much larger fraction of pairs of individuals as related that are reported as unrelated, suggesting it may be biased toward detecting higher levels of IBD sharing than the other methods.

Noting that the SAMAFS consist of admixed Mexican American individuals, we examined the accuracy results among the allele frequency-based methods, several of which account for population structure. While IBD segment-based methods generally have the best performance and do not directly account for population structure, inferring IBD segments is computationally demanding, and considering the performance of more efficient allele frequency-based methods is of interest. Among all these methods, PC-Relate has the highest accuracy across all levels of relatedness, and it accounts for population structure using principal components (PCs) inferred from a set of samples with low relatedness (Conomos *et al.* 2016). However, PREST-plus has only slightly lower performance than PC-Relate even though it does not account for population structure. PREST-plus implements a hidden Markov model that enables it to leverage linkage signals to identify regions that are likely to be IBD between samples (Sun and Dimitromanolakis 2014). Therefore, although PREST-plus does not explicitly detect IBD segments, it leverages similar signals to the IBD segment-based approaches, which might enable it to be less susceptible to biases caused by ignoring the effects of population structure. Relatedness estimation that ignores population structure in admixed samples can produce either a positive or negative bias (Conomos *et al.* 2016). Consistent with this, PLINK infers many sample pairs to be more related than they are reported to be, and, at the same time, infers substantial fractions of fourth- through seventh-degree pairs as unrelated. KING also dramatically underestimates relatedness, presumably because it assumes that all samples derive from one of several homogeneous populations, a model that is inappropriate for recently admixed samples (Manichaikul *et al.* 2010). We also examined results from the version of KING that assumes a single homogeneous population, and its accuracy profile more closely resembles that of PLINK (data not shown).

Because the relatedness within SAMAFS has the potential to confound methods that characterize population structure (Conomos *et al.* 2015), we further analyzed the performance of several methods using a data set consisting of the SAMAFS samples together with a diverse set of HapMap individuals (International HapMap 3 Consortium *et al.* 2010) (Figure S4 and Supplemental Note in File S1). This combined data set yields inferences of sample ancestry proportions that are strongly correlated with those inferred in a reduced data set that has only low-level relatedness (Supplemental Note in File S1). Using this sample, the accuracies of both REAP and RelateAdmix improve significantly, suggesting that either high levels of relatedness or limited ability to discriminate the ancestral populations in the admixed-only SAMAFS data adversely affected the initial inference. Based on this augmented analysis, REAP and RelateAdmix have closer accuracies to that of PC-Relate yet remain somewhat less accurate (Figure S4 and Supplemental Note in File S1). The accuracies of PC-Relate and of KING are quite similar between the two analyses, with the exception that PC-Relate has improved accuracy for seventh-degree relatives in the larger sample. Given this improvement and the fact that PC-Relate is the highest performing allele frequency-based method overall, we tested it further by varying its input parameters and the kinship values it uses to detect the set of individuals it uses to infer PCs. All these PC-Relate runs resulted in similar accuracies except for different rates of inferred seventh-degree relatives (Figure S5 and Supplemental Note in File S1); the variation in seventh-degree relatedness inference may be due to stochastic factors and the relatively small numbers of these relatives in the data set.

Besides considerations related to detecting population structure, the presence of many relatives in SAMAFS may lead to biased allele frequency estimates. Furthermore, haplotype phasing and therefore IBD inference accuracy might be greater than would be achieved in a sample composed mostly of unrelated individuals. To ensure the performance results presented here also apply to analyses of nonpedigree data sets, we identified a set of only distantly related individuals using FastIndep (Abraham and Diaz 2014) and merged these samples with pairs of related individuals to form 1000 data sets (Supplemental Note in File S1). Each reduced data set contains at most one related pair of samples from any distinct SAMAFS pedigree, limiting the potential for bias. When classifying sample pairs included in at least one reduced data set, PLINK’s inference accuracy differs by <3% for the first through fifth relatedness degrees compared to the full data set (Figure S2 in File S1), suggesting that allele frequency biases are small and only minimally affect inference accuracy. To test the IBD detection methods, we increased the sample size of these reduced data sets by further merging 580 HapMap samples (Supplemental Note in File S1). Results from running the IBD segment-based methods on these data sets show a reduction in accuracy that ranges between 0 and 9.6% for first- through fifth-degree relatives, indicating that relatedness in SAMAFS may affect the inference accuracy (Figure S3 in File S1). Yet the results are still consistent with those of the larger analysis as the IBD segment-based methods generally have higher performance than allele frequency-based methods. This is true even in the reduced data sets that have no more than 1204 samples and therefore are subject to a nontrivial rate of phasing error (Browning and Browning 2011b).

In comparison to previous method evaluations, our results show some notable differences. For example, using real data from 30 pedigrees, ERSA reported lower accuracies for first- through sixth-degree relatives than we observe (Li *et al.* 2014), with differences ranging from 8.9 to nearly 21%. We believe this is attributable to differences in sample size, as the ERSA analysis considered only 304 individuals compared to 2485 here. This, in addition to the decreased accuracy of IBD segment-based methods in the reduced data sets described above, indicates that sample size can have a dramatic impact on the quality of IBD segment-based methods. Thus, smaller studies may wish to use allele frequency-based methods such as PC-Relate or, for nonadmixed individuals, KING-robust, which in fact considers data from each sample pair separately rather than estimating allele frequencies from the full data (Manichaikul *et al.* 2010). The authors of PC-Relate (Conomos *et al.* 2016) find that KING and PLINK each tend to both overestimate and underestimate relatedness when analyzing admixed individuals, which is consistent with our results. They also report that PC-Relate generally outperforms REAP and RelateAdmix, matching our findings even after we incorporate additional HapMap individuals to aid detection of population structure (Supplemental Note in File S1). To our knowledge, other evaluations of relatedness inference approaches have not included methods that directly detect IBD segments, and our results indicate that these are promising methods to apply in this setting.

As current methods provide only moderate accuracy when classifying third- through seventh-degree relatives, we evaluated the potential for increasing performance by combining inference results from the top three programs: ERSA, GERMLINE, and Refined IBD. We first used an approach that calls the degree of relatedness for a pair only when all three programs unanimously agree on the relatedness degree, providing no classification for other pairs (3012 relative pairs and 632,615 reported unrelated pairs are unclassified). In comparison to the most accurate method’s performance in each degree class, the inference accuracy using this strategy increases only slightly for related pairs (+0.01, +0.13, +2.6, +1.5, +3.4, +2.2, and +1.1%, respectively, for first-through seventh-degree), but increases by 9.0% for unrelated pairs. This indicates a high level of discordance among the inferred relatedness status for a large fraction of pairs that are reported as unrelated. Many of these unrelated pairs must therefore have borderline inferences, and indeed most methods infer a sizeable fraction as only eighth-degree relatives (Figure 1). We also considered a majority vote between the three programs, discarding cases in which all three programs inferred a different degree (only five relative pairs had such variable inferences while 110,848 pairs reported as unrelated are so discrepant). With this approach, there is a slight decrease in performance overall (−0.04, −0.6, −1.3, −0.7, −0.2, −2.3, and 0% for first- through seventh-degree relatives and +1.6% for unrelated samples). These results suggest that while there is room for improvement in the specificity of relatedness inference methods, dramatic accuracy gains are likely to be achieved only with novel approaches and not composites of current methods. Of interest in this regard are recently developed methods that combine information across related individuals to infer a pedigree structure and/or improve relatedness accuracy (Staples *et al.* 2014, 2016; Ko and Nielsen 2017). Importantly, each of these methods relies on a pairwise relatedness approach, highlighting the continued relevance of pairwise inference methodologies even as new methods arise for addressing multi-way relatedness inference.

As an application of these findings, we leveraged the high accuracy of IBD segment-based methods to explore pairs of samples inferred to be closely related but reported as unrelated in the SAMAFS data set. We used the top-performing methods, ERSA, GERMLINE, and Refined IBD, to characterize unreported relatives. These three methods all infer a small number of first- through third-degree relationships that connect individuals from different pedigrees within SAMAFS (Figure S7 and Supplemental Note in File S1). Overall, we found six pairs of pedigrees with at least five sample pairs between them that the methods unanimously infer to have first- through third-degree relatedness. Additionally, these three methods agree on the inference of 235 and 744 pairs of fourth- and fifth-degree relatives between the pedigrees (data not shown), and suggest instances of reported first- and second-degree relatives likely to have the reverse relatedness class or to have much lower relatedness (Supplemental Note and Table S3 in File S1). These results highlight the necessity of checking reported relationships and for unreported relatedness among samples in all cohorts. They also indicate that there can be sizeable numbers of unknown relatives across a range of relatedness degrees even in well-studied samples.

Important factors for determining which analysis method to use in a study are its accuracy and its computational demands, and the runtimes of the methods evaluated here vary over several orders of magnitude (Table 2). PLINK is the fastest program with a runtime of only 18.1 sec, while the IBD segment-based methods require up to 64 compute days in total (parallelized across 16 cores in our analyses). In general, we observe a trade-off between runtime and accuracy, with the top-performing methods being those that require the largest compute time, and with PLINK being one of the least accurate methods. Given the uniformly high accuracy of all methods for inferring first- and second-degree relatives, applications that are focused only on identifying close relatives have the option of using an efficient allele frequency-based method such as PLINK or PC-Relate to perform inference, the latter being an accurate program that is more computational intensive than PLINK but much faster than IBD segment-based methods. A further consideration is the ethnic group of the analysis cohort. PLINK and KING have biased results for distant relatives in the admixed SAMAFS data we focus on, but are expected to perform well in homogeneous populations or, for KING, collections of unadmixed samples from multiple homogeneous populations. On the other hand, for applications in which the aims include locating more distant relatives, the use of IBD segment-based methods should produce improved results. Although beyond the scope of this paper, recently developed methods for phasing extremely large samples (Loh *et al.* 2016) should improve upon the computational requirements of several methods (GERMLINE, ERSA, and HaploScore) and extend their utility to much larger data sets than the one we consider here.

We have presented a detailed comparison of state-of-the-art relatedness inference methods using thousands of pairs of individuals that range from first- to seventh-degree relatives as well as numerous sample pairs that are reported to be unrelated. All the methods we assessed reliably identify first- and second-degree relatives (accuracy ∼92–99%), but their accuracy falls precipitously when classifying third- to seventh-degree relatives. This is unsurprising given the increased coefficient of variation as well as greater skewness in the proportion of genome shared as the meiotic distance between two relatives increases (Hill and Weir 2011). Despite these challenges, several IBD segment-based methods infer relatedness correct to within one degree of the reported relationship at a rate of ≥76.7% for all relationship degrees (Figure 1). Misreported or unknown relationships in the SAMAFS data set likely explain some of the inference errors, particularly since even some confidently inferred first-degree relationships were likely misreported as a more distant relationship or as unrelated (Figure S7 and Table S3 in File S1). We find that IBD segment-based methods outperform other approaches for more distantly related pairs, though notably these packages require substantially more compute time to run (Table 2). While the precise performance results presented here are specific to the SAMAFS sample, we find that reducing the sample size still produces similar results, with methods that leverage IBD segments generally having greater accuracy than other approaches. Therefore, the results presented here should be generalizable to moderate and large-scale studies and indicate overall properties of pairwise relationship inference methodologies: approaches that use IBD segments outperform other methods for third-degree and more distant relatives; and the specificity of the inferences, even in a data set where phase accuracy may be relatively high, are limited for all but the closest relatives.

## Data Availability

The SAMAFS sample data are available on dbGaP under accession numbers phs000847 and phs001215. A script to extract pairwise IBD1 and IBD2 proportions from the output of Refined IBD can be found at https://github.com/MonicaRamstetter/bakeoff.

## Supplementary Material

Supplemental material is available online at www.genetics.org/lookup/suppl/doi:10.1534/genetics.117.1122/-/DC1.
