# Supplementary material for: Benchmarking Relatedness Inference Methods with Genome-Wide Data from Thousands of Relatives
Source: Genetics. 2017 Jul 26;207(1):75–82. doi: 10.1534/genetics.117.1122 (PMC5586387; doi:10.1534/genetics.117.1122)
Supplement: Supplementary file 1 [file 75FileS1.pdf]

Supplemental Materials

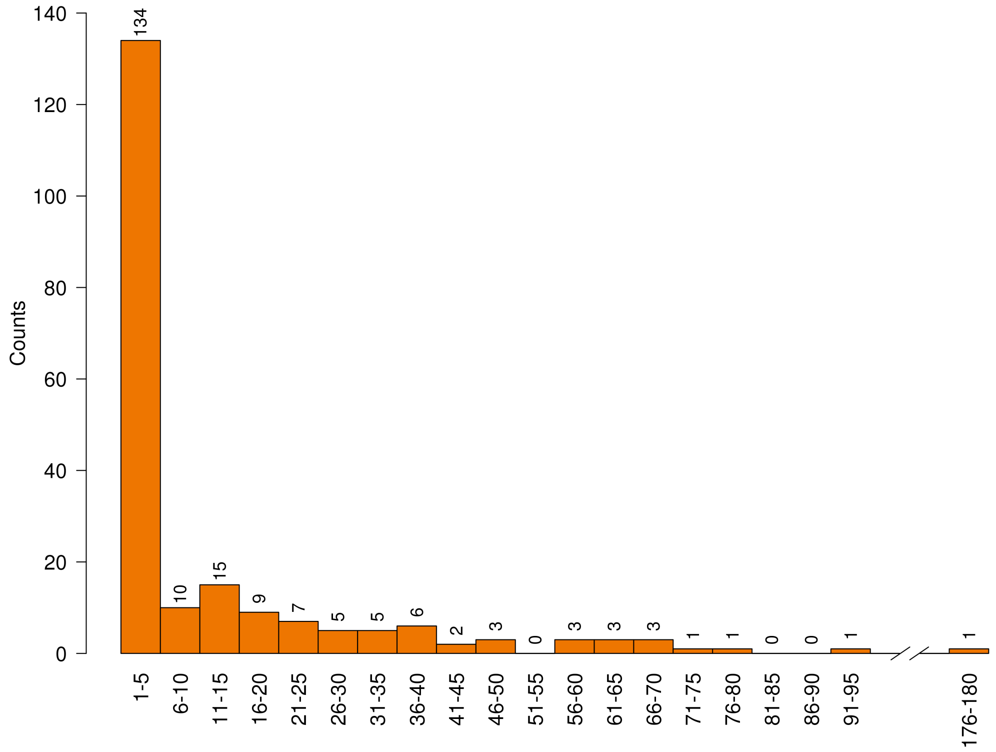

Supplemental Figure 1: Histogram showing the number of SAMAFS pedigrees that have the given number of genotyped samples.

	Values that map to degree:				Expected			
Degree	ϕ	$k^{(0)}$	Relationship	# Meioses	$k^{(0)}$	$k^{(1)}$	$k^{(2)}$	ϕ
0	$> \frac{1}{2^{3/2}}$	< 0.1	Monozygotic (MZ) twin	0	0	0	1	$\frac{1}{2}$
1	$(\frac{1}{2^{5/2}}, \frac{1}{2^{3/2}}]$	< 0.1	Parent-child	1	0	1	0	$\frac{1}{2^2}$
	$(\frac{1}{2^{5/2}}, \frac{1}{2^{3/2}}]$	$[0.1, 0.365)$	Full sibling (not MZ twin)	2 (×2)	1/4	1/2	1/4	$\frac{1}{2^2}$
2	$(\frac{1}{2^{7/2}}, \frac{1}{2^{5/2}}]$	$[0.365, 1 - \frac{1}{2^{3/2}})$	Grandparent-grandchild	2	1/2	1/2	0	$\frac{1}{2^3}$
			Avuncular	3 (×2)	1/2	1/2	0	$\frac{1}{2^3}$
			Double-cousin	4 (×4)	9/16	3/8	1/16	$\frac{1}{2^3}$
			Half-sibling	2	1/2	1/2	0	$\frac{1}{2^3}$
3	$(\frac{1}{2^{9/2}}, \frac{1}{2^{7/2}}]$	$[1 - \frac{1}{2^{3/2}}, 1 - \frac{1}{2^{5/2}})$	First cousin	4 (×2)	3/4	1/4	0	$\frac{1}{2^4}$
			Great-grandparent	3	3/4	1/4	0	$\frac{1}{2^4}$
			Grand-avuncular	4 (×2)	3/4	1/4	0	$\frac{1}{2^4}$
			Half-avuncular	3	3/4	1/4	0	$\frac{1}{2^4}$
4	$(\frac{1}{2^{11/2}}, \frac{1}{2^{9/2}}]$	$[1 - \frac{1}{2^{5/2}}, 1 - \frac{1}{2^{7/2}})$	First cousin once removed	5 (×2)	7/8	1/8	0	$\frac{1}{2^5}$
			Great-great-grandparent	4	7/8	1/8	0	$\frac{1}{2^5}$
			Great-grand-avuncular	5 (×2)	7/8	1/8	0	$\frac{1}{2^5}$
			Half-grand-avuncular	4	7/8	1/8	0	$\frac{1}{2^5}$
5	$(\frac{1}{2^{13/2}}, \frac{1}{2^{11/2}}]$	$[1 - \frac{1}{2^{7/2}}, 1 - \frac{1}{2^{9/2}})$	First cousin twice removed	6 (×2)	15/16	1/16	0	$\frac{1}{2^6}$
			Second cousin	6 (×2)	15/16	1/16	0	$\frac{1}{2^6}$
			GGG-grandparent	5	15/16	1/16	0	$\frac{1}{2^6}$
6	$(\frac{1}{2^{15/2}}, \frac{1}{2^{13/2}}]$	$[1 - \frac{1}{2^{9/2}}, 1 - \frac{1}{2^{11/2}})$	Second cousin once removed	7 (×2)	31/32	1/32	0	$\frac{1}{2^7}$
7	$(\frac{1}{2^{17/2}}, \frac{1}{2^{15/2}}]$	$[1 - \frac{1}{2^{11/2}}, 1 - \frac{1}{2^{13/2}})$	Second cousin twice removed	8 (×2)	63/64	1/64	0	$\frac{1}{2^8}$
			Third cousin	8 (×2)	63/64	1/64	0	$\frac{1}{2^8}$
8	$(\frac{1}{2^{19/2}}, \frac{1}{2^{17/2}}]$	$[1 - \frac{1}{2^{13/2}}, 1 - \frac{1}{2^{15/2}})$	Third cousin once removed	9 (×2)	127/128	1/128	0	$\frac{1}{2^9}$

Supplemental Table 1: Degrees of relatedness with the ranges of kinship coefficients (ϕ) and genome proportions inferred to be IBD0 ($k^{(0)}$) that map to that degree. Includes example relationships for each degree and the corresponding number of meioses that separate a pair of individuals with that relationship. Number of meioses with text (×2) and (×4) correspond, respectively, to samples that are related along two (e.g., full siblings) and four lines of descent, with the indicated meiotic distance on all lines. Also lists the proportions of the genome that are expected to be IBD0, IBD1, and IBD2 between samples that have each relationship, denoted $k^{(0)}$, $k^{(1)}$, and $k^{(2)}$, respectively; and the corresponding expected kinship coefficient ϕ. Table does not include all possible relationship types for these degrees of relatedness.

Supplemental Note

SAMAFS data and quality control procedures

Our analysis focuses on SNP array data from the San Antonio Mexican American Family Studies[1–3] (SAMAFS). The 2,490 samples were genotyped via one of the following Illumina arrays: the Human660W, Human1M, Human1M-Duo, or both the HumanHap500 and the HumanExon510S array which together provide roughly the same content as the Human1M array. We began by using data that had quality control measures carried out in a prior study[4]. In brief, sites with non-Mendelian errors were set to missing and we used BWA[5] v0.7.5a-r405 to map the SNP array probe sequences to human reference sequence GRCh37. Only SNPs with probe sequences that aligned with no mismatches at exactly one genomic position and that do not align to any other location with either zero or one mismatches were kept. We omitted SNPs for

which any of the following was true: (1) multiple probes aligned to the same location (we retained only one SNP for any location), (2) dbSNP reported either more than two variants or had non-simple alleles (i.e., not A/C/G/T), (3) the raw genotype data had differing alleles as compared to those reported in the manifest files, (4) the manifest file listed SNP alleles that differed from those in dbSNP at the aligned location, (5) dbSNP listed the SNP as having multiple locations or as 'suspected', (6) the SNP location is outside the 'accessible genome' as reported by the 1000 Genomes Project[6], (7) the site occurs in a region that is segmentally duplicated with a Jukes-Cantor K-value of <2%, or (8) the site occurs within a total of 17 Mb of the genome that received excess reads aligned using 1000 Genome Project data[7].

Following these procedures, we filtered the dataset to include SNPs with less than 2% missingness and individuals with less than 10% missingness. This resulted in data for a total of 2,485 individuals at 521,184 SNPs that overlap between the two types of arrays and are of high quality. To run methods that require independent SNPs, we used the PLINK command `--indep-pairwise 1000 25 0.25` which uses a sliding window method that considers blocks of 1,000 SNPs and removes SNPs with $r^2 > 0.25$, afterward shifting the window by 25 SNPs. This process yielded a total of 140,314 SNPs after linkage disequilibrium (LD) was filtered. We explore the effect of different filtering thresholds below in the "Varying linkage disequilibrium filters" section.

For the ADMIXTURE[8] analyses described below, we merged the above LD-pruned SAMAFS dataset with HapMap phase 3 samples[9] and again filtered to include SNPs with less than 2% missingness from the combined dataset. This resulted in a sample with 102,527 SNPs.

After an initial analysis, we discovered several pairs of individuals reported as unrelated but inferred to be first or second degree relatives based on the output from Refined IBD. When these individuals are in the same SAMAFS pedigree, relatedness degrees between their descendants can be inflated relative to that implied by the reported relationships. In order to reduce this effect, we excluded from consideration reported pairs of relatives in which both samples descend from one of the unexpected relatives. Specifically, for a given pair of inferred but unreported relatives from the same pedigree, (ind_1, ind_2), we found the sets D_1 and D_2 of all descendants of ind_1 and ind_2, respectively. Then for each $d_1 \in D_1$ and $d_2 \in D_2$, we removed from the set of relatives to be analyzed each pair (d_1, d_2), as well as all pairs of the forms (ind_1, d_2) and (d_1, ind_2). This resulted in the removal of 2,618 pairs of individuals: 3, 80, 322, 625, 763, 554, 120, and 151 pairs reported as second through eighth degree relatives and unrelated, respectively.

Some reported relationships in the dataset have an expected kinship coefficient that does not map to a specific degree of relatedness. For example, three-quarter-siblings share one parent in common and have unshared parents that are first degree relatives (such as full siblings); their expected kinship coefficient is intermediate between first and second degree relatives, and as a result their degree of relatedness is unclear. We omit all such relatives from the analysis, with a total of 91 pairs removed.

Although monozygotic (MZ) twins have a degree of relatedness of 0, for the analyses in this paper, we combine the 10 reported pairs of MZ twins with the set of first degree relatives and call all individuals with estimated kinship $\phi > \frac{1}{2^{5/2}}$ as first degree relatives.

Effects of the SAMAFS relatedness structure on inference quality

As the SAMAFS data consist of numerous large families, allele and haplotype frequencies estimated from the sample may be biased, potentially affecting the inference results in a way that is not representative of the methods' accuracy in other datasets. To assess how severely this may impact the results of the allele frequency-based methods on all of SAMAFS, we tested PLINK[10] on datasets composed primarily of individuals with fairly low level relatedness. To generate these sample sets, we first determined a set of distantly related individuals using FastIndep[11], a program that uses estimated kinship coefficients and a maximum allowed relatedness threshold to identify a set of individuals in which no pairwise relatedness exceeds the given threshold. For pairs reported as unrelated, we use the kinship coefficients from PLINK, and for pairs reported as related, we use the expected kinship coefficient (Supplemental Table 1) value for that pair. We input these kinship coefficients to FastIndep with the relatedness threshold set to 0.015, which is roughly the expected kinship coefficient for fifth degree relatives. This produced a set of 529 individuals,

Degree	Number of Pairs
1	4,510
2	5,999
3	7,030
4	5,991
5	2,496
Unrelated	1,997,907
Total	2,023,933

Supplemental Table 2: Number of pairs of individuals that passed quality filters, are reported to have first through fifth degree relatedness, and which we included in at least one of the 1,000 datasets composed primarily of individuals with PLINK-estimated kinship less than 0.015. (See the "Effects of reducing relatedness in input data" section.) Pairs counted as unrelated for this analysis are from distinct pedigrees. We include reported MZ twins with the set of first degree relatives.

denoted as L, in which all sample pairs are expected or estimated to share no more than 3% of their genome IBD. We note that PLINK is somewhat biased in inferring relatedness and identifies a non-trivial proportion of samples that are reported to be unrelated as fifth degree or closer relatives (Figure 1). Therefore, using PLINK kinship estimates should provide an aggressive filter against potential relatedness in these sample sets. Next, we created 1,000 datasets containing the base set of samples L merged with no more than one randomly selected pair of related individuals from each SAMAFS pedigree, resulting in a total of 26,026 pairs of fifth degree or closer relatives, and nearly two million pairs from distinct pedigrees so reported as unrelated (Supplemental Table 2). When adding a related pair of individuals to the dataset, we checked if either of the individuals is reported to be a fifth degree or closer relative of a sample in L, and in that case, removed the sample in L from the dataset. Finally, we ran PLINK on each of the 1,000 datasets and show performance accuracy results in comparison to running PLINK on the full SAMAFS dataset in Supplemental Figure 2. While some differences exist between the two analyses, the accuracy results differ by less than 3% for all considered relatedness classes, indicating that the relatedness structure within SAMAFS has little affect on allele frequency estimates and resulting relatedness measures.

In the presence of multiple copies of various haplotype segments that stem from the numerous relatives in the full SAMAFS dataset, phasing and therefore IBD accuracy may be inflated compared to non-pedigree samples. This may increase the inference quality of IBD segment-based programs (which utilize either internal phasing models or pre-phased data) compared to the other programs. To assess the performance of the IBD segment-based methods in a setting with relatively outbred data, we again used datasets comprised mostly of individuals with low relatedness. Specifically, starting with the 1,000 datasets with the base set of samples contained in L as outlined above, we merged genotypes from 580 HapMap phase 3 individuals (83 individuals of African ancestry in Southwest USA [ASW], 165 Utah residents with Northern and Western European ancestry from the CEPH collection [CEU], 77 samples of Mexican ancestry in Los Angeles, California [MXL], 88 Toscani in Italia individuals [TSI], and 167 Yoruba in Ibadan, Nigeria samples [YRI]) in order to increase the sample size. This provides a baseline level of phase accuracy that should be achievable for most studies as all these datasets contain between 1,127−1,204 individuals. Results from this analysis are presented in Supplemental Figure 3. The accuracy of the IBD segment-based methods does drop for higher degrees of relatedness in the reduced datasets compared to all of SAMAFS, in some cases by as much as 9.6%. In this case the performance of IBD segment methods and allele frequency methods are more similar, suggesting that for smaller datasets, phasing errors can reduce the efficacy of IBD segment methods for inferring relatedness. Still, the IBD segment-based methods are comparable to or more accurate than the allele frequency methods even in this setting. Moreover, for larger datasets where it is possible to achieve phase accuracy at the megabase-scale[12], the results from the full dataset indicate that IBD segment-based methods provide greater accuracy than allele frequency-based methods for inferring relatives with third degree or more distant relatedness.

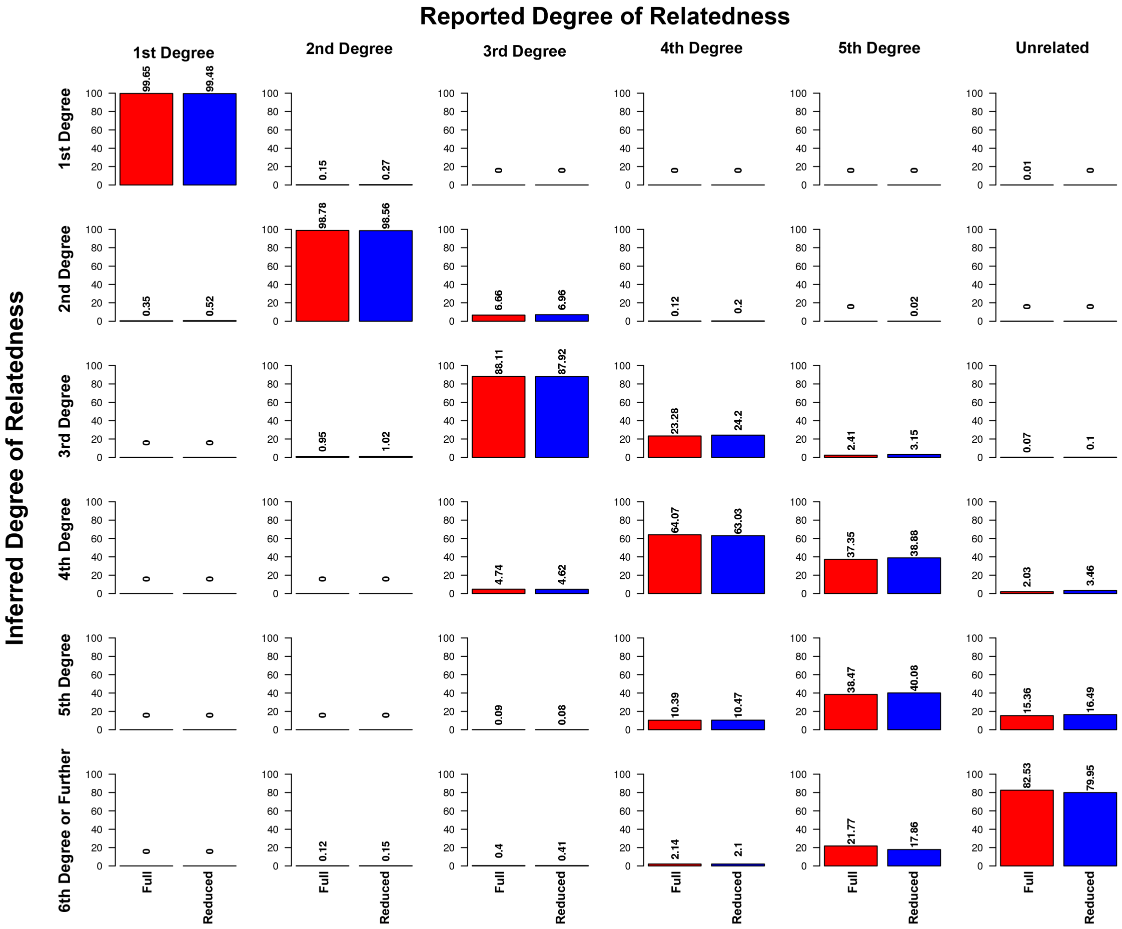

Supplemental Figure 2: Accuracy results from PLINK run on the entire SAMAFS dataset (labeled "Full"), and from PLINK run on 1,000 reduced datasets composed mostly of individuals with PLINK-derived kinship estimates of less than 0.015 (labeled "Reduced"). When a pair of relatives is present in more than one reduced dataset, we selected results from an arbitrary dataset to determine accuracy. We combined results for pairs inferred to have sixth degree or more distant relatedness in one class for this analysis.

Addressing population structure while including data from diverse samples

Samples that have admixed ancestry can confound relatedness estimation methods due to some individuals having more or less similar population-level ancestry, thus inducing an increased or decreased correlation in genotypes among admixed samples that may or may not be recently related[13,14]. While methods such as REAP[13] and RelateAdmix[15] adjust for admixture, they rely on the output of model-based ancestry inference methods such as ADMIXTURE[8] which have difficulty distinguishing between ancestral populations and more recent relatedness among samples[14].

We sought to determine whether adding genotypic variance corresponding to the populations relevant to the ancestry of the SAMAFS samples would enable ADMIXTURE to reliably estimate ancestral population proportions despite the relatedness in the full dataset. To that end, we generated a dataset containing the entire (LD-pruned) SAMAFS sample together with 372 unrelated HapMap individuals. These HapMap individuals are a subset of the 580 individuals described above (including samples with African, European, and Native American ancestry pertinent to SAMAFS), but with samples filtered out by FastIndep using previously estimated kinship coefficients[16] as input and a filtering threshold of 0.015. We then ran ADMIXTURE on this dataset with $K = 3$. Next, we ran ADMIXTURE with $K = 3$ on another dataset containing the 372

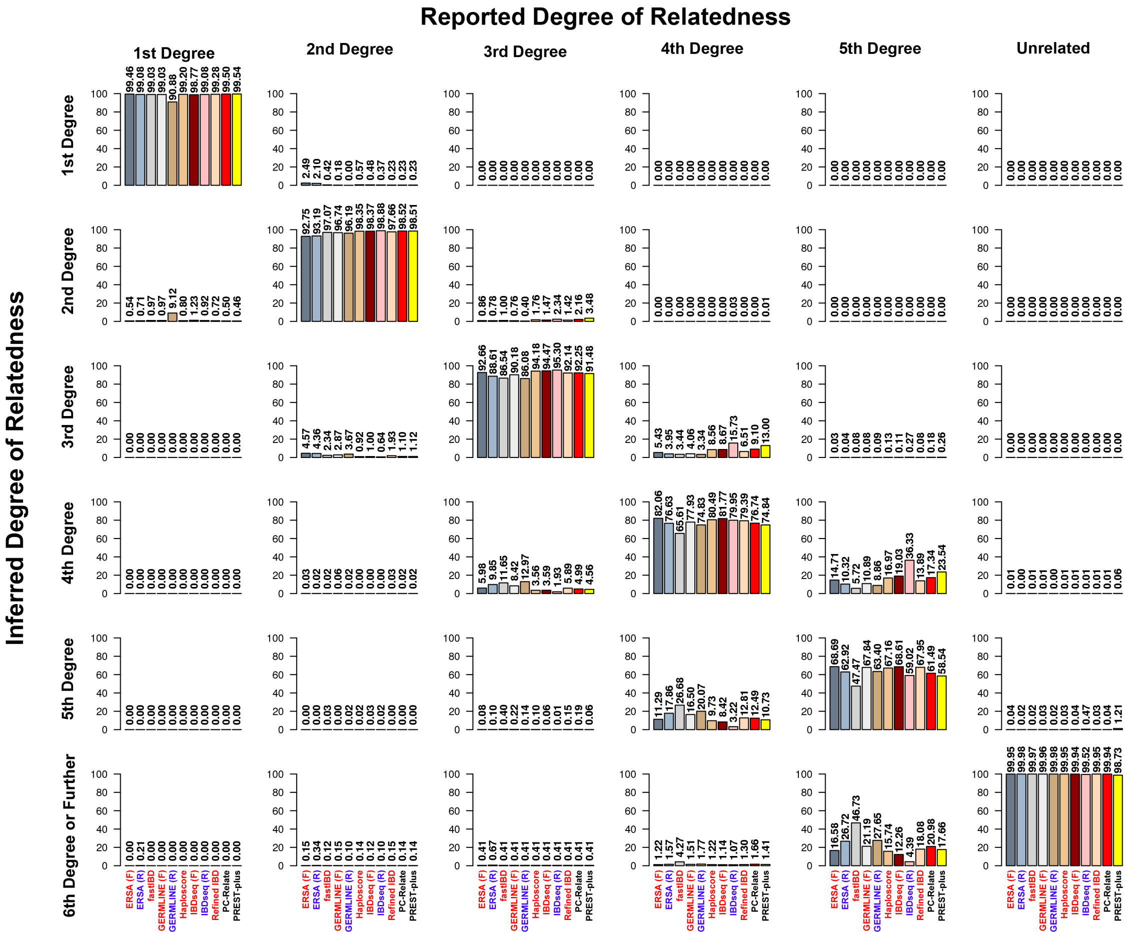

Supplemental Figure 3: Accuracy results from the full dataset for all IBD segment-based methods, PC-Relate, and PREST-plus along with results from running ERSA, GERMLINE, and IBDseq on 1,000 reduced datasets composed mostly of individuals with inferred kinship values of less than 0.015. Results from programs run on both types of data are indicated with a label "(F)" and red text for the full dataset and "(R)" and blue text for the reduced datasets. The accuracies in all cases are for pairs of samples that were included in at least one reduced dataset so that the results are directly comparable between data types. We report sample pairs inferred as sixth degree or more distant together here.

unrelated HapMap samples and the set L (above) containing 529 SAMAFS samples inferred by PLINK to have kinship less than 0.015. As this latter dataset has little relatedness structure, ADMIXTURE should readily infer the samples' ancestral proportions from African, European, and Native American populations. Consistent with this, we located the inferred ancestry components likely to correspond to these three groups using individuals from the HapMap YRI, CEU, and MXL populations in the two different ADMIXTURE runs. We then computed correlations separately for each component between the set L of 529 SAMAFS samples contained in both datasets. These correlations are extremely high at >0.97 for all three populations, indicating that the output from ADMIXTURE run on all of SAMAFS together with the 372 unrelated HapMap individuals reliably infers population-level ancestry proportions.

Building on this analysis, we used the ADMIXTURE results from the dataset containing the full SAMAFS and unrelated HapMap samples, extracting only the ancestry estimates for SAMAFS, in order to examine whether the accuracy of REAP and RelateAdmix improve using these ancestry estimates. Additionally, we ran KING and PC-Relate (the other methods that address population structure) on this same combined

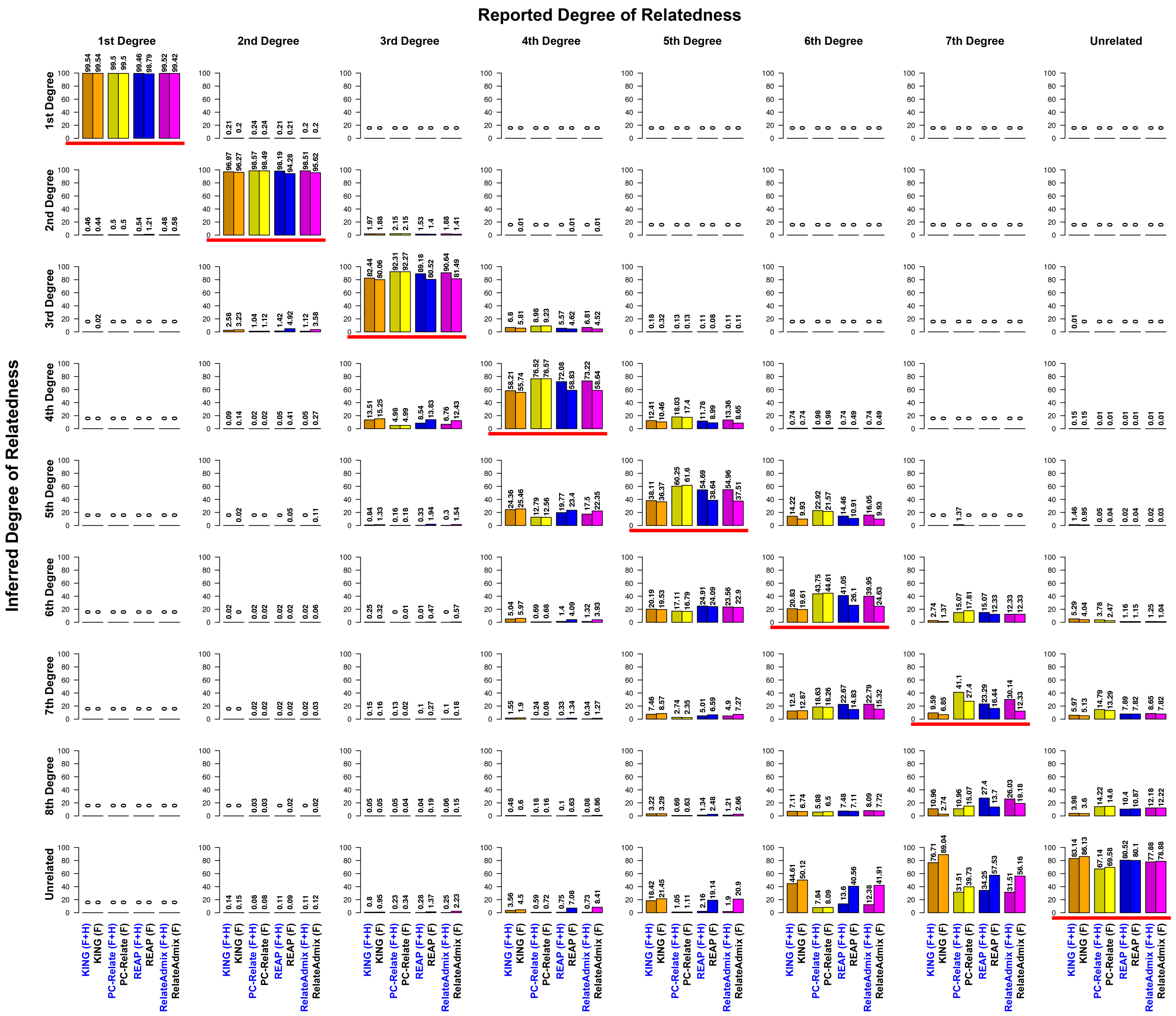

Supplemental Figure 4: Accuracies from running KING, PC-Relate, REAP, and RelateAdmix on: a dataset containing all of SAMAFS and unrelated HapMap individuals, with a label "(F+H)" in blue text; and on the full SAMAFS sample, with an added label "(F)" in black text. Red horizontal bars under a bar plot indicate that the corresponding inferences agree with the reported relationships.

dataset to compare their performance using this more diverse sample to that from the SAMAFS-only data. As shown in Supplemental Figure 4, the accuracy of REAP and RelateAdmix increase, indicating that the ADMIXTURE results improve when using the augmented dataset. In contrast, the performance of KING and PC-Relate are generally consistent in both analyses. As PC-Relate identifies a set of individuals with little relatedness and uses these to infer principal components (PCs), it is less susceptible to confounding due to relatedness in the input data, and appears not to require aids to distinguish shared variation due to population ancestry from recent relatedness. Notably, in the case of seventh degree relatives PC-Relate's

accuracy increases to 41.1% compared to 27.4% in the original dataset. While this increase is striking, our analysis includes fewer than 100 seventh degree relatives, and it is unclear to what extent this result may be due to stochastic factors. Overall, PC-Relate performs well in both settings, and indeed outperforms REAP and RelateAdmix regardless of whether we include HapMap samples with SAMAFS.

Additional analyses of PC-Relate

We further examined the potential for improving accuracy in PC-Relate by (1) using different methods to produce the kinship coefficients given to PC-Relate as input, testing PLINK and Refined IBD as well as the recommended method KING[17] (as used in the main analysis); (2) changing the relatedness threshold that determines the set of samples used to infer PCs, varying this between the default of excluding fourth degree relatives or closer to excluding seventh degree relatives or closer; and (3) iterating PC-Relate by using its own inferred kinship coefficients as input to a second round inference, using either KING or Refined IBD as the initial input, an approach the authors suggested previously[17]. As shown in Supplemental Figure 5, all tested scenarios produce similar accuracy, with sizeable variation only observed in seventh degree relative inference, potentially driven by noisy results for this smaller number of distant relatives. We conclude that the results in Figure 1 are representative of PC-Relate's optimal performance in SAMAFS.

Varying linkage disequilibrium filters

For methods that are designed to handle only independent SNPs, LD filtering of the genotype data is necessary, but the parameters used in filtering have the potential to affect inference quality. We consider the accuracy of inferring relatedness using genotypes with r^2 up to 0.05, 0.1, 0.2, and our original threshold of 0.25. Using the original SAMAFS dataset, we filtered using the `--indep-pairwise 1000 x` option in PLINK, where `x` is the upper bound for r^2. This resulted in datasets with 23,761, 57,198, and 114,541 SNPs for r^2 set to 0.05, 0.1, and 0.2, respectively. Supplemental Figure 6 shows the rates for inferring sample pairs to have the same degree of relatedness as reported in SAMAFS as well as one degree higher and lower. In general, changes in accuracy for inferring individuals to have the reported relatedness degree are minimal. Some larger differences exist for seventh degree relative inference, but as previously noted, this is a smaller set of relative pairs and differences may be due to random fluctuations. No clear trend exists among the methods, as some have improved performance for lower r^2 thresholds, while some perform best with the highest value.

Samples with inferred relatedness different from that reported in SAMAFS

We sought to identify relationships that are reported as either first degree, second degree, or unrelated, but are inferred to have a substantially different degree of relatedness. Using unanimous agreement from ERSA, GERMLINE, and Refined IBD to locate confidently inferred relationships, we located several pairs of samples with such differences. For inferred relationships that differ by more than one degree from the reported relationship (e.g., reported as second degree but inferred as sixth degree), we assumed that the inference is valid as this is unlikely to occur due to data errors or statistical fluctuations. For relationships that are inferred to differ by only one degree from the reported relationship, we further required that either: (1) the discrepant relatedness call be supported by a consistent call involving at least one other sample (example follows); (2) in cases of reported siblings inferred to be second degree relatives, that their IBD2 proportion be less than $\frac{1}{2^{5/2}}$; or (3) in cases of reported half-sibling pairs inferred to be first degree relatives, that their IBD2 proportion be greater than $\frac{1}{2^{5/2}}$. As an example of an inference supported by another sample, given a set of three or more reported siblings, if the methods infer a pair of siblings as likely second degree relatives (presumably half-siblings), we checked that one of the other siblings also support a second degree relatedness inference involving one of the two original samples to ensure consistency. We used results from Refined IBD to quantify IBD2 levels. Note that the expected proportion of IBD2 between full siblings is $\frac{1}{4}$, and we used $\frac{1}{2^{5/2}}$ as the cutoff for confirming full vs. half-sibling calls.

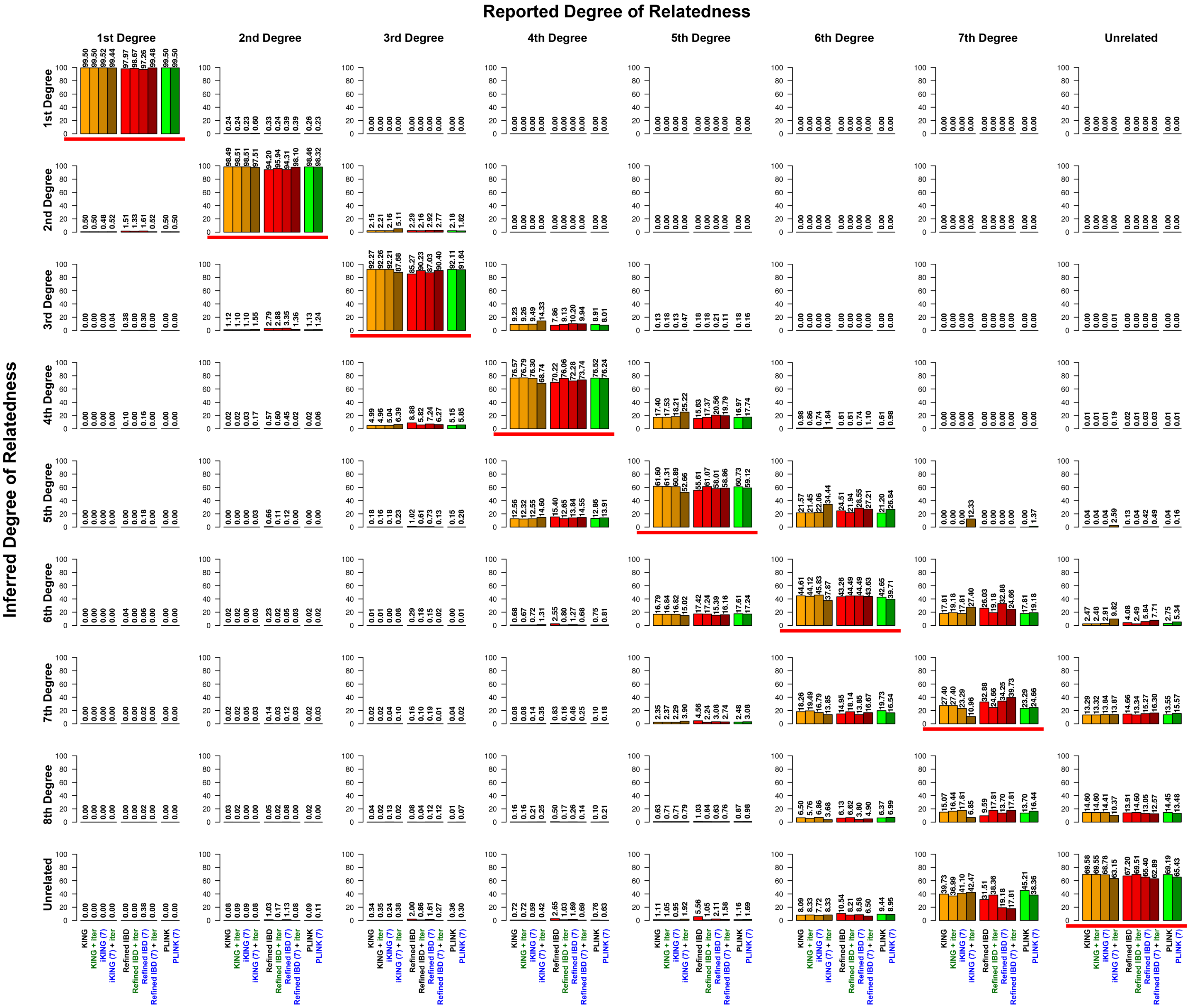

Supplemental Figure 5: Inference results from running PC-Relate with different input and parameter settings. Labels indicate the method used to supply input kinship coefficients—KING, Refined IBD, or PLINK—that PC-Relate uses to identify samples with which it infers PCs. Labels in green with "+ iter" text correspond to results from iterating by rerunning PC-Relate using its own kinship estimates as input for a second round of inference. Labels in blue with "(7)" are from running PC-Relate with a threshold set to exclude seventh degree or closer relatives rather than the default of excluding fourth degree or closer relatives. Red horizontal bars under a bar plot indicates that the corresponding inferences agree with the reported relationships.

The IBD2 levels of two reported half-siblings from two pedigrees were greater than that seen for most half-siblings but less than typical for full siblings, and appear to be best explained as being a less commonly described class of relatives known as three-quarter-siblings defined earlier (see "SAMAFS data and quality control procedures"). Individuals with this class of relatedness share non-trivial proportions of IBD2 but at

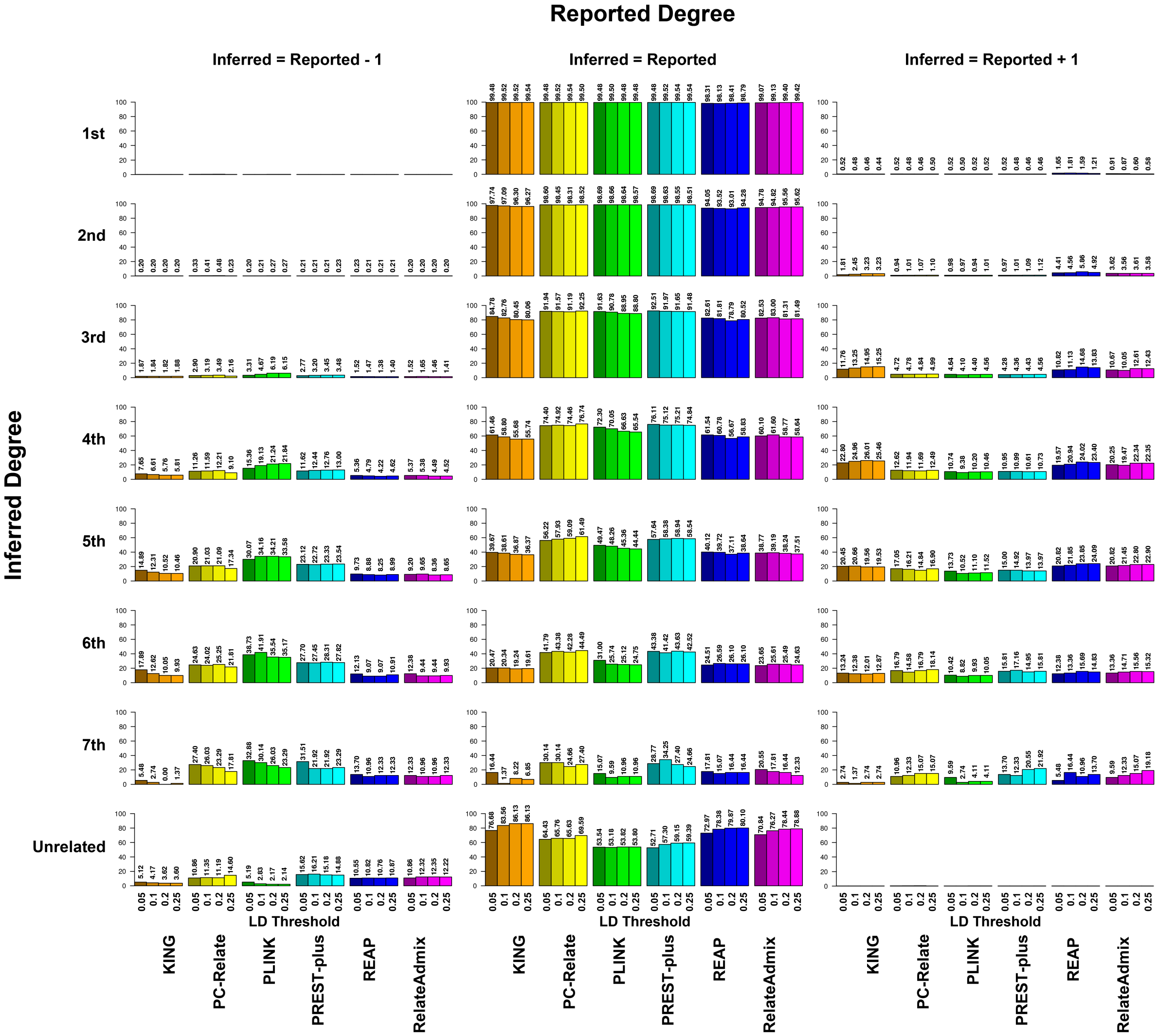

Supplemental Figure 6: Accuracies of KING, PC-Relate, PLINK, REAP, and RelateAdmix using four different LD filtering thresholds: 0.05, 0.1, 0.2, and 0.25. Depicts the proportion of sample pairs inferred to have the same degree of relatedness as reported as well as inferred to have one higher and one lower relatedness degree.

a lower level than for full siblings. For the potential three-quarter-siblings we identified, we did not have genotype data for one of the fathers in both cases and therefore could not validate whether the fathers were siblings. As the degree of relatedness for such pairs is ambiguous, and because other factors may explain these findings, we do not count these pairs as inconsistent. As shown in Supplemental Table 3, the majority of pairs in the dataset have inferred relatedness consistent with the reported relationships. However, we find a few discrepancies, including unrelated individuals inferred to be closely related (Supplemental Figure 7), and even some second degree relatives inferred to have sixth degree or more distant relatedness. This latter group could be explained by sample swaps or possibly by the individuals being adopted.

		Inferred		
		1st	2nd	6th Degree or Further
**Reported**	1st	**4,919**	23	0
	2nd (HS)	10	**380**	6
	2nd (A)	2	**4,725**	3
	2nd (GP)	0	**933**	0
	2nd (DC)	0	**8**	0
	Unrelated	5	23	**3,051,455**

Supplemental Table 3: Pairs of relationships that are confidently inferred using unanimous agreement from three programs and further checks described in the text (for some discrepant relationships) in SAMAFS. (HS) are half-sibling pairs, (A) are avuncular pairs, (GP) are grandparent-grandchild pairs, and (DC) are double cousin pairs. Bold numbers indicate agreement between reported and inferred relationships. We only count pairs whose relationships are unanimously agreed upon by the methods and, when discrepant, could be verified as probable misreports using the described checks.

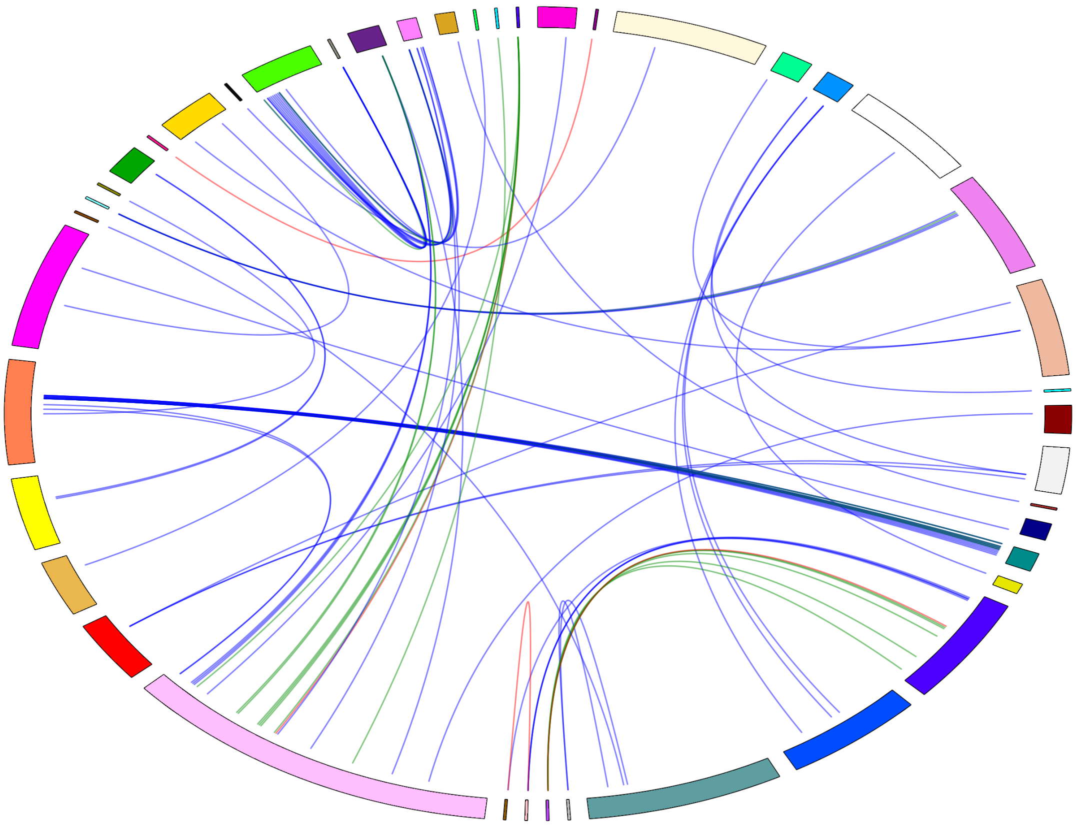

Supplemental Figure 7: Relationships discovered between individuals from different SAMAFS pedigrees. Bands on the perimeter of the elliptical plot indicate distinct pedigrees within SAMAFS with band size proportional to the number of individuals in the pedigree. Curves between two bands correspond to discovered relative pairs with curve color indicating the degree of relatedness: red for first degree, green for second degree, and blue for third degree. Points where the curves end correspond to specific individuals, and a single point may have multiple curves running to it, indicating several relationships between that individual and others in the dataset.

Method testing details

Details on how we ran each of the methods appears below. Note that these descriptions apply to the inference results presented in Figure 1. We performed several additional analyses using altered input or parameters to the methods that are described above. In all cases, we provided methods with genotype data only and removed all reported family relationships.

ERSA: We ran ERSA using output from GERMLINE run with the recommended parameters of `-err_het 2 -err_hom 1 -min_m 1`.

fastIBD: Following recommended practices[18], we ran fastIBD using the default settings 10 times using a different seed each time and took the union of the called IBD segments from the multiple runs as our final set of IBD regions.

GERMLINE: We phased the data using Beagle 4.1 and ran GERMLINE on the inferred haplotypes with the parameters recommended for ERSA (`-err_het 2 -err_hom 1 -min_m 1`) as well as with the `-haploid` option to reduce over-calling of IBD segments and to enable detection of IBD2 regions.

HaploScore: We ran HaploScore with the default settings using the same GERMLINE output as for ERSA.

IBDseq: We ran IBDseq with the default settings.

KING: We ran KING with the `--kinship` flag in order to run KING-robust (i.e., the version that allows for population structure) using the LD-filtered dataset.

PC-Relate: We used the LD-filtered dataset and the pairwise relatedness matrix inferred by KING-robust as input to PC-AiR[14] (as recommended by the authors[17]). PC-Relate expects input PCs inferred using PC-AiR, and the latter outputs 20 PCs by default. We examined the eigenvalues and scatter plots of these PCs and determined that three PCs is a suitable number to use for capturing the variation in SAMAFS; three PCs also gives nearly identical results to using 20 PCs (not shown). We used the defaults for all other settings.

PLINK: We used the `--genome full` option in PLINK to infer relatedness using the LD-filtered dataset.

PREST-plus: We used the LD-pruned data and default settings with the `-aped -wped` flags. These flags, respectively, instruct the program to calculate IBD estimates across and within pedigrees.

REAP: We ran ADMIXTURE with $K = 3$ on the LD-pruned dataset to provide ancestry estimates to REAP. We then ran REAP with the flags `-k 3 -t 0`, where `-k 3` corresponds to analyzing with three ancestral populations and `-t 0` instructs REAP to output all estimated pairwise kinship coefficients.

Refined IBD: As recommended by the authors[19], we ran Refined IBD three times using the default settings and different seeds for each run. Reported regions of IBD were considered IBD2 when two reported segments on different haplotypes overlapped. After finding IBD2 regions in each individual run, we took the union of these IBD2 regions across all three runs as our final set of IBD2 regions. Regions reported as IBD in any of the three methods but not included in the set of IBD2 regions constitute the final set of IBD1 regions.

RelateAdmix: We provided RelateAdmix the same ADMIXTURE results as REAP and ran it on the LD-pruned dataset.

Method inclusion criteria

Our analysis considers only pairwise inference methods, and does not include those that combine information across pairs of related individuals such as PRIMUS[20], PADRE[21], AKT[22], and a recently developed simulated annealing method[23]. We further omit methods that focus only on inferring first and second degree relationship types such as RELPAIR[24] and RelCla[25]. Several papers describe statistical approaches for inferring relatedness[26–30] but to our knowledge no released program exists that we could evaluate. A number of other methods[31–37] are either not available for download, provided incomplete documentation, failed to

complete the analysis due to apparent bugs, and/or required the purchase of a license to run, and we did not include these in our study.

References

[1] Braxton D Mitchell, Candace M Kammerer, John Blangero, Michael C Mahaney, David L Rainwater, Bennett Dyke, James E Hixson, Richard D Henkel, R Mark Sharp, Anthony G Comuzzie, et al. Genetic and environmental contributions to cardiovascular risk factors in Mexican Americans. *Circulation*, 94(9):2159–2170, 1996.

[2] Ravindranath Duggirala, John Blangero, Laura Almasy, Thomas D Dyer, Kenneth L Williams, Robin J Leach, Peter O'Connell, and Michael P Stern. Linkage of type 2 diabetes mellitus and of age at onset to a genetic location on chromosome 10q in Mexican Americans. *American Journal of Human Genetics*, 64(4):1127–1140, 1999.

[3] Kelly J Hunt, Donna M Lehman, Rector Arya, Sharon Fowler, Robin J Leach, Harald HH Göring, Laura Almasy, John Blangero, Tom D Dyer, Ravindranath Duggirala, et al. Genome-wide linkage analyses of type 2 diabetes in Mexican Americans. *Diabetes*, 54(9):2655–2662, 2005.

[4] Amy L Williams, Giulio Genovese, Thomas Dyer, Nicolas Altemose, Katherine Truax, Goo Jun, Nick Patterson, Simon R Myers, Joanne E Curran, Ravi Duggirala, et al. Non-crossover gene conversions show strong GC bias and unexpected clustering in humans. *eLife*, 4:e04637, 2015.

[5] Heng Li and Richard Durbin. Fast and accurate short read alignment with Burrows–Wheeler transform. *Bioinformatics*, 25(14):1754–1760, 2009.

[6] 1000 Genomes Project Consortium and others. An integrated map of genetic variation from 1,092 human genomes. *Nature*, 491(7422):56–65, 2012.

[7] Giulio Genovese, Robert E Handsaker, Heng Li, Eimear E Kenny, and Steven A McCarroll. Mapping the human reference genome's missing sequence by three-way admixture in latino genomes. *The American Journal of Human Genetics*, 93(3):411–421, 2013.

[8] David H Alexander, John Novembre, and Kenneth Lange. Fast model-based estimation of ancestry in unrelated individuals. *Genome Research*, 19(9):1655–1664, 2009.

[9] International HapMap 3 Consortium and others. Integrating common and rare genetic variation in diverse human populations. *Nature*, 467(7311):52–58, 2010.

[10] Christopher C Chang, Carson C Chow, Laurent CAM Tellier, Shashaank Vattikuti, Shaun M Purcell, and James J Lee. Second-generation PLINK: rising to the challenge of larger and richer datasets. *Gigascience*, 4(1):1, 2015.

[11] Kuruvilla Joseph Abraham and Clara Diaz. Identifying large sets of unrelated individuals and unrelated markers. *Source code for biology and medicine*, 9(1):1, 2014.

[12] Po-Ru Loh, Pier Francesco Palamara, and Alkes L Price. Fast and accurate long-range phasing in a UK Biobank cohort. *Nature Genetics*, 2016.

[13] Timothy Thornton, Hua Tang, Thomas J Hoffmann, Heather M Ochs-Balcom, Bette J Caan, and Neil Risch. Estimating kinship in admixed populations. *American Journal of Human Genetics*, 91(1):122–138, 2012.

[14] Matthew P Conomos, Michael B Miller, and Timothy A Thornton. Robust inference of population structure for ancestry prediction and correction of stratification in the presence of relatedness. *Genetic Epidemiology*, 39(4):276–293, 2015.

[15] Ida Moltke and Anders Albrechtsen. RelateAdmix: a software tool for estimating relatedness between admixed individuals. *Bioinformatics*, 30(7):1027–1028, 2014.

[16] Eric L Stevens, Joseph D Baugher, Matthew D Shirley, Laurence P Frelin, and Jonathan Pevsner. Unexpected relationships and inbreeding in HapMap phase III populations. *PLOS ONE*, 7(11):e49575, 2012.

[17] Matthew P Conomos, Alexander P Reiner, Bruce S Weir, and Timothy A Thornton. Model-free estimation of recent genetic relatedness. *American Journal of Human Genetics*, 98(1):127–148, 2016.

[18] Brian L Browning and Sharon R Browning. A fast, powerful method for detecting identity by descent. *American Journal of Human Genetics*, 88(2):173–182, 2011.

[19] Brian L Browning and Sharon R Browning. Improving the accuracy and efficiency of identity-by-descent detection in population data. *Genetics*, 194(2):459–471, 2013.

[20] Jeffrey Staples, Dandi Qiao, Michael H Cho, Edwin K Silverman, Deborah A Nickerson, Jennifer E Below, University of Washington Center for Mendelian Genomics, et al. PRIMUS: rapid reconstruction of pedigrees from genome-wide estimates of identity by descent. *American Journal of Human Genetics*, 95(5):553–564, 2014.

[21] Jeffrey Staples, David J Witherspoon, Lynn B Jorde, Deborah A Nickerson, Jennifer E Below, Chad D Huff, University of Washington Center for Mendelian Genomics, et al. PADRE: Pedigree-aware distant-relationship estimation. *The American Journal of Human Genetics*, 99(1):154–162, 2016.

[22] Rudy Arthur, Ole Schulz-Trieglaff, Anthony J Cox, and Jared O'Connell. AKT: ancestry and kinship toolkit. *Bioinformatics*, 33(1):142–144, 2017.

[23] Amy Ko and Rasmus Nielsen. Composite likelihood method for inferring local pedigrees. *bioRxiv*, page 106492, 2017.

[24] Michael P Epstein, William L Duren, and Michael Boehnke. Improved inference of relationship for pairs of individuals. *American Journal of Human Genetics*, 67(5):1219–1231, 2000.

[25] Chaozhi Zheng, Mary K Kuhner, and Elizabeth A Thompson. Joint inference of identity by descent along multiple chromosomes from population samples. *Journal of Computational Biology*, 21(3):185–200, 2014.

[26] EA Thompson. The estimation of pairwise relationships. *Annals of Human Genetics*, 39(2):173–188, 1975.

[27] Brook G Milligan. Maximum-likelihood estimation of relatedness. *Genetics*, 163(3):1153–1167, 2003.

[28] Amy D Anderson and Bruce S Weir. A maximum-likelihood method for the estimation of pairwise relatedness in structured populations. *Genetics*, 176(1):421–440, 2007.

[29] William G Hill and Ian MS White. Identification of pedigree relationship from genome sharing. *G3: Genes— Genomes— Genetics*, pages g3–113, 2013.

[30] Manuel García-Magariños, Thore Egeland, Ignacio López-de Ullibarri, Nils L Hjort, and Antonio Salas. A parametric approach to kinship hypothesis testing using identity-by-descent parameters. *Statistical applications in genetics and molecular biology*, 14(5):465–479, 2015.

[31] Sofia Kyriazopoulou-Panagiotopoulou, Dorna Kashef Haghighi, Sarah J Aerni, Andreas Sundquist, Sivan Bercovici, and Serafim Batzoglou. Reconstruction of genealogical relationships with applications to Phase III of HapMap. *Bioinformatics*, 27(13):i333–i341, 2011.

[32] Eric L Stevens, Greg Heckenberg, ED Roberson, Joseph D Baugher, Thomas J Downey, and Jonathan Pevsner. Inference of relationships in population data using identity-by-descent and identity-by-state. *PLOS Genetics*, 7(9):e1002287, 2011.

[33] Brenna M Henn, Lawrence Hon, J Michael Macpherson, Nick Eriksson, Serge Saxonov, Itsik Pe'er, and Joanna L Mountain. Cryptic distant relatives are common in both isolated and cosmopolitan genetic samples. *PLoS One*, 7(4):e34267, 2012.

[34] Hong Li, Gustavo Glusman, Chad Huff, Juan Caballero, and Jared C Roach. Accurate and robust prediction of genetic relationship from whole-genome sequences. *PLOS ONE*, 9(2):e85437, 2014.

[35] Catherine A Ball, Mathew J Barber, Jake Byrnes, Peter Carbonetto, Kenneth G Chahine, Ross E Curtis, Julie M Granka, Eunjung Han, Eurie L Hong, Amir R Kermany, et al. Ancestry DNA matching white paper. 2016.

[36] Hyokyeong Lee and Liang Chen. Inference of kinship using spatial distributions of SNPs for genome-wide association studies. *BMC genomics*, 17(1):372, 2016.

[37] Anna Shcherbina, Darrell O Ricke, Eric Schwoebel, Tara Boettcher, Christina Zook, Johanna Bobrow, Martha Petrovick, and Edward Wack. KinLinks: Software toolkit for kinship analysis and pedigree generation from hts datasets. *bioRxiv*, page 046938, 2016.
